# Harnessing Cyanobacteria’s Bioactive Potential: A Sustainable Strategy for Antioxidant Production

**DOI:** 10.3390/microorganisms12010175

**Published:** 2024-01-16

**Authors:** Teresa De Rosa, Maria Ponticelli, Roberta Teta, Vittorio Carlucci, Luigi Milella, Germana Esposito, Valeria Costantino

**Affiliations:** 1 The Blue Chemistry Lab, Department of Pharmacy, University of Naples Federico II, Via Domenico Montesano 49, 80131 Napoli, Italy; teresa.derosa@unina.it (T.D.R.); roberta.teta@unina.it (R.T.); 2 Department of Science, Università Degli Studi Della Basilicata, V.le dell’Ateneo Lucano 10, 85100 Potenza, Italy; maria.ponticelli@unibas.it (M.P.); vittorio.carlucci@unibas.it (V.C.); luigi.milella@unibas.it (L.M.)

**Keywords:** cyanobacteria, *Anabaena*, natural products, mass spectrometry, molecular network, antioxidant, algal blooms, OSMAC approach, unsaturated diacylglycerols

## Abstract

Unsaturated diacylglycerols are a class of antioxidant compounds with the potential to positively impact human health. Their ability to combat oxidative stress through radical scavenger activity underscores their significance in the context of preventive and therapeutic strategies. In this paper we highlight the role of *Anabaena flos-aquae* as a producer of unsaturated mono and diacylglycerols, and then demonstrate the antioxidant activity of its methanolic extract, which has as its main components a variety of acylglycerol analogues. This finding was revealed using a sustainable strategy in which the One Strain Many Compounds (OSMAC) cultivation in microscale was coupled with a bioinformatic approach to analyze a large dataset of mass spectrometry data using the molecular networking analyses. This strategy reduces time and costs, avoiding long and expensive steps of purification and obtaining informative data on the metabolic composition of the extracts. This study highlights the role of *Anabaena* as a sustainable and green source of novel bioactive compounds.

## 1. Introduction

Cyanobacteria, commonly known as “blue-green algae”, represent some of the earliest photosynthetic prokaryotes on Earth. They showcase remarkable adaptability, thriving in a wide range of habitats, including thermal springs, subglacial environments, and subterranean rock formations in arid regions. Cyanobacteria can be categorized based on their morphology as unicellular, colonial, or multicellular filamentous, with cell diameters spanning from 1 to 100 μm. Their outer membrane, consisting of peptidoglycan and lipopolysaccharide, forms a complex multilayered structure designed to protect the organism in challenging environments.

Similar to higher plants, cyanobacteria are photoautotrophic organisms with the capability to convert light into chemical energy while releasing oxygen. To achieve this, they utilize a diverse array of pigments, encompassing chlorophyll-a (a green pigment), phycobilins, such as phycocyanin (PC) (a blue pigment) and phycoerythrin (PE) (a red pigment), in addition to carotenoids. This pigmentation system allows cyanobacteria to efficiently capture and harness light energy for their photosynthetic processes.

Elevated temperatures and an excess of nutrients, particularly nitrogen and phosphorus, can create favorable conditions for the proliferation of cyanobacteria in aquatic ecosystems, giving rise to the formation of harmful algal blooms (HABs). These blooms have detrimental effects on multiple facets of the aquatic environment, affecting public health, the quality of drinking water, recreational activities, and the tourism industry [1].

Indeed, during the bloom phenomenon, there is a significant and dramatic increase in the production of toxins, leading to various management challenges for local governments and municipalities. The toxic compounds generated by cyanobacteria can be broadly categorized into three groups: hepatotoxins, neurotoxins, and lipopolysaccharide (LPS) endotoxins. These toxins pose a risk to human health and have been implicated in numerous cases of animal poisonings.

Conversely, cyanobacteria possess the capability to synthesize a diverse range of secondary metabolites, each with distinct biological properties. This synthesis is influenced by the intricate interplay between microorganisms and their environment, considering factors such as the availability of oxygen and chemical energy. Notable among these secondary metabolites are polysulfated polysaccharides with antiviral effects, polyunsaturated fatty acids, β-carotene, and other pigments that exhibit antioxidant properties. Additionally, mycosporine-like amino acids (MAAs) and scytonemin function as photoprotectants, while sterols demonstrate antimicrobial properties. This multifaceted repertoire of secondary metabolites highlights the adaptability and versatility of cyanobacteria in responding to their ecological surroundings.

Human applications of secondary metabolites produced by cyanobacteria extend to diverse medical and environmental purposes. In the realm of environmental science, the potential applications of these secondary metabolites are manifold. Cyanobacteria-derived secondary metabolites have been found to exert positive effects on plant and fish health, prompting their utilization in agricultural and aquacultural practices. The recognition of the beneficial impacts of these metabolites highlights the promising contributions of cyanobacteria to environmental sustainability and ecosystem health Inizio modulo [2].

The quest for novel antioxidant compounds holds exceptional significance in modern nutraceutical research and development. Antioxidants are pivotal in counteracting oxidative stress, a process linked to various health disorders and the aging process. The increasing interest in the search for natural antioxidant compounds is propelled by apprehensions regarding the use of preservatives and the potential toxicity associated with existing synthetic antioxidants. This pursuit reflects a commitment to exploring safer and more sustainable alternatives to enhance health and well-being. Indeed, several synthetic antioxidants, despite their efficacy, may be accompanied by drawbacks, such as toxicity or undesirable side effects. Turning to natural sources for antioxidants presents an opportunity to broaden the spectrum of available compounds, diminishing reliance on synthetic alternatives and potentially enabling the discovery of safer options for therapeutic applications.

The interest in investigating antioxidant activity in cyanobacteria stems from their rich repertoire of compounds, including carotenoids and polyphenols. A substantial body of literature has delved into the antioxidant activity of cyanobacteria originating from both freshwater and terrestrial habitats. This exploration reflects a keen interest in leveraging the natural diversity of cyanobacterial compounds for their potential health-promoting and therapeutic benefits [3].

Since 2013, our research group has been dedicated to the study of cyanobacterial metabolites. This journey commenced with the discovery of smenamides A and B, unique hybrid peptide/polyketide compounds featuring a dolapyrrolidinone unit from the holobiome of the sponge *Smenospongia aurea*. The structural resemblances to cyanobacterial metabolites hint at an intriguing interplay between the sponge host and its cyanobacterial symbionts.

Motivated by this discovery, we initiated an extensive investigation involving laboratory-cultivated cyanobacterial strains. Our overarching objectives encompass not only advancing scientific knowledge but also exploring the practical applications and sustainable technologies that can be derived from the capabilities of cyanobacteria. This research endeavor reflects our commitment to unravelling the potential of cyanobacteria in both fundamental understanding and real-world applications.

Among others, the OSMAC approach [4], an acronym for One Strain Many Compounds, constitutes a strategic methodology in the realms of natural product discovery and drug development. This approach is grounded in the understanding that the biosynthetic capabilities of microorganisms, including bacteria and fungi, can be modulated by diverse environmental and cultural conditions. By methodically altering these conditions, it becomes possible to elicit the production of a myriad of secondary metabolites from a single microbial strain. This systematic variation in environmental conditions aims to unlock the diverse secondary metabolites that the strain could potentially produce, providing valuable insights for further exploration in natural product discovery and drug development.

In our unwavering pursuit of novel secondary metabolites with potential pharmacological activity, adhering to the OSMAC approach, we harnessed the biosynthetic capabilities of the cyanobacterium *Anabaena flos-aquae* UTEX 1444 cultivated under six distinct concentrations of iron (III) [5].

Cyanobacteria exhibit a pronounced dependence on substantial amounts of iron, serving as an indispensable cofactor for fundamental metabolic processes, such as photosynthesis, respiration, and nitrogen fixation [6] This need for iron surpasses that of non-photosynthetic organisms. To adapt and survive in conditions of limited iron availability, cyanobacteria have developed diverse strategies, one of which involves the production of siderophores. Siderophores are compounds with a low molecular weight (ranging from 400 to 1000 kDa) and are secreted by microorganisms and plants. They are renowned for being among the most potent chelators of Fe^3+^.

The data obtained from our experiments unveiled the presence of several analogues of synechobactin in the extracts from cultures grown in absence of iron [5] The confirmation of synechobactin presence was achieved through the utilization of HRMS/MS analysis facilitated by molecular networking. This investigation provides valuable insights into the adaptive mechanisms of cyanobacteria under varying iron conditions, emphasizing the significance of siderophores, particularly synechobactins, in their response to limited iron availability. The application of this tool enabled us to determine the metabolic content of the extracts at an early stage of the analysis, circumventing the need for extensive purification procedures. This contributes to a sustainable strategy by reducing the use of solvents and shortening detection times.

In this work, the five organic extracts (#1 Methanol Extract, #2 Methanol-Chloroform Extract, #3 Chloroform Extract, #4 Butanol Extract and #5 Aqueous phase) of *Anabaena flos-aquae* UTEX 1444 grown in iron deficiency conditions were tested for antioxidant activity.

The active methanolic extract was profiled through HRMS/MS based molecular networking, aiming to uncover new and promising antioxidant compounds within the metabolic repertoire of the cyanobacterial strain.

In this manuscript, we demonstrate the application of our sustainable strategy for investigating the metabolomic composition of biomass extracts and their potential bioactivity. Our findings reveal the presence of unsaturated mono and diacylglycerols, identified as responsible for the observed antioxidant activity in the MeOH extract. Furthermore, the study underscores the promising potential of *Anabaena* as a valuable bioresource for nutraceuticals.

## 2. Materials and Methods

### 2.1. Cultivating and Harvesting Anabaena flos-aquae UTEX 1444

The *Anabaena flos-aquae* UTEX 1444 strain, obtained from the collection of Professor A. Pollio (Department of Biology, University of Naples Federico II, Napoli, Italy), was grown in modified fresh water BG-11 media (FW BG-11) containing per liter of solution: 1 mg of Na_2_EDTA, 6 mg of citric acid, 500 mg of NaNO_3_, 40 mg of K_2_HPO_4_·3H_2_O, 75 mg of MgSO_4_·7H_2_O, 26.4 mg of CaCl_2_, 17.1 mg of NaCO_3_, 250 μL of NiSO_4_(NH_4_)_2_SO_4_·6H_2_O (0.1 mM stock solution), 100 μL of Na_2_SeO_4_ (0.1 mM stock solution), and 1 mL of Nitsch Solution (100 mL of Nitsch’s solution contains: 50 μL of concentrated H_2_SO_4_, 0.228 mg of MnSO_4_·H_2_O, 50 mg of ZnSO_4_·7H_2_O, 1.59 mg of CuSO_4_, 2.5 mg of Na_2_MoO_4_·2H_2_O, 50 mg of H_3_BO_3_, and 13.5 mg of CoCl_2_·6H_2_O), fortified with 100 μL of vitamin mix (100 mL of 10× solution contains: 100 mg of nicotine acid, 10 mg of PABA, 1 mg of biotin, 251 mg of thiamine hydrochloride, 1 mg of vitamin B12, 1 mg of folic acid, 1 mg of inositol, and 100 mg of calcium pantothenate). Specifically, the culture did not contain any source of iron (the standard BG11 medium contains 6 mg ferric ammonium citrate per liter). The culture was kept in an incubator at room temperature (18–25 °C) for one month. The specimen was examined using an optical microscope (Motic Panthera C2 microscope, Barcelona, Spain). Subsequently, it underwent vortexing, sonication (ARGO Lab Digital Ultrasonic Cleaner, Model-DU 32, Carpi, Italy) at 20 KHz for 10 min at 23 °C, and centrifugation (HERMLE Labortechnik, Model-Z36HK, Wehingen, Germany) at 10,000 rpm for 5 min at 25 °C to separate solid pellet from the liquid supernatant. The pellet was then subjected to extraction using organic solvents as outlined below: (1#) MeOH (100%, 0.3 L), (2#) MeOH/CHCl_3_ (1:1, 0.3 L), and (3#) CHCl_3_ (100%, 0.3 L). Supernatant was extracted with BuOH, undergoing partitioning twice to obtain a (4#) BuOH phase and a (5#) H_2_O phase.

All the extracts underwent paper filtration and were concentrated under vacuum, resulting in: 18.4 mg (#1), 5.8 mg (2#), 4.1 mg (3#), 33.5 mg (4#), and 300 mg (5#). Each extract was resuspended in MeOH (100%, 5 mg/mL) for the subsequent LC-HRMS and LC-HRMS/MS analyses.

### 2.2. Antioxidant Assays

#### 2.2.1. Total Phenolic Content Assay

The TPC was assessed through the Folin–Ciocalteau method. In brief, 75 μL of the diluted extract was combined with distilled water (425 μL), Folin–Ciocalteau reagent (500 μL), and Na_2_CO_3_ (10% *w*/*v*) (500 μL). This mixture was vortexed and left in darkness at room temperature for an hour. Subsequently, the absorbance was measured at 723 nm using a UV-Vis spectrophotometer (SPECTROstarNano, BMG Labtech, Ortenberg, Germany). Gallic Acid served as the reference antioxidant standard, and the data were presented as mg of Gallic Acid Equivalent (GAE) per gram of dried extract (DW). TPC assessments for all extracts were conducted in triplicate [7].

#### 2.2.2. 2,2-Diphenyl-1-Picrylhydrazyl Assay

The radical-scavenging activity was evaluated by 2,2-Diphenyl-1-picrylhydrazyl (DPPH) assay. Each extract (50 µL) was diluted to 200 µL of DPPH methanol solution (100 mM) in a 96-well plate. The mixture was shaken vigorously and left in a dark place after 30 min. The absorbance was read at 515 nm using a UV-Vis spectrophotometer (SPECTROstarNano, BMG Labtech, Ortenberg, Germany). Trolox was used as a reference antioxidant standard and the results were expressed as mg Trolox Equivalent/100 g of fresh weight (mg TE/100 g FW). DPPH for all extracts was carried out in triplicate [8].

#### 2.2.3. Ferric Reducing Antioxidant Power Assay

The FRAP reagent, formulated as buffer A (300 mM sodium acetate anhydrous in acetic acid, pH 3.6), buffer B (10 mM TPTZ in 40 mM HCl), and buffer C (20 mM FeCl_3_·6H_2_O in distilled water) in a ratio of 10:1:1, was prepared. A total of 225 μL of the FRAP mixture was combined with 25 μL of various extract dilutions in a 96-well plate, followed by a 40-min incubation at 37 °C in the absence of light. For the blank sample, 225 μL of FRAP reagent was mixed with 25 μL of methanol. The absorbance of the solution was assessed at 593 nm. Trolox served as the reference antioxidant standard, and the results were presented as milligrams of Trolox equivalents per gram of dried extract (mg TE/g DW). The FRAP assay was conducted in triplicate for all samples [7].

#### 2.2.4. β-Carotene Bleaching Assay

The β-carotene-linoleic acid bleaching method (BCB) evaluated the antioxidant activity. β-Carotene solution (0.6 mg of β-carotene dissolved in 0.6 mL of chloroform), linoleic acid (45 μL), and Tween 20 (200 mg) were mixed. Chloroform was removed using a rotary evaporator at room temperature. Distilled water (50 mL) was added with oxygen. Then, 950 μL of the emulsion was transferred into several tubes containing 50 μL of sample (the final concentration for all tested samples was 0.1 mg/mL) or methanol as blank. BHT was used as a positive control. The tubes were placed at 50 °C for 3 h. The absorbance was measured at 470 nm at 0′, 30′, 60′, 90′, 120′, 150′, and 180′. Each sample was carried out in triplicate. Results were expressed as a percentage of β-carotene bleaching inhibition and calculated as follows: (Aβ-carotene after 180 min/A initial β-carotene) × 100 (AA%) [9].

#### 2.2.5. Relative Antioxidant Capacity Index (RACI)

The Relative Antioxidant Capacity Index (RACI) was determined by combining the results obtained from TPC, DPPH, FRAP, and BCB. Specifically, by using this index and thus comparing data expressed with different measurement units, each dataset is converted into a standard score. In the field of statistics, a standard score (also known as normal score or z-) is a non-dimensional number calculated by subtracting the mean from the raw data and multiplying it by the standard deviation. The standard score was thus determined by employing Excel software (2010, Microsoft, Redmond, WA, USA) and applying the following equation:

$$\frac{\left(x-\mu \right)}{\sigma }$$

*x* indicates the raw data, *µ* the mean, and *σ* the standard deviation. Subtracting the mean makes the distribution centered, and dividing the difference by the standard deviation makes the distribution normalized [10]. A high RACI score suggests significant relative antioxidant activity [11].

### 2.3. LC-HRMS and LC-HRMS/MS Analysis

LC-HRMS and LC-HRMS/MS experiments were conducted using a Thermo LTQ Orbitrap XL high-resolution ESI mass spectrometer (Thermofisher, Waltham MA, USA), coupled with a Thermo U3000 HPLC system (Thermofisher, Waltham MA, USA). The LC system comprised a solvent reservoir, an in-line degasser, a binary pump, and a refrigerated autosampler. A Kinetex C18 column (50 × 2.10 mm) with a particle size of 5 µm was utilized and maintained at room temperature. The elution was performed at a rate of 200 µL/min with a gradient of H_2_O (supplemented with 0.1% HCOOH) and MeOH. The gradient program consisted of 10% MeOH for 3 min, followed by a transition from 10% to 100% MeOH over 30 min, and a subsequent 100% MeOH elution for 7 min. The volume injected was set at 5 µL. Mass spectra were acquired in positive ion detection mode. MS parameters were a spray voltage of 4.8 kV, a capillary temperature of 285 °C, a sheath gas rate of 32 units N_2_ (ca. 150 mL/min), and an auxiliary gas rate of 15 units N_2_ (ca. 50 mL/min). Data were collected in the data-dependent acquisition (DDA) mode, in which the five most intense ions of each full-scan mass spectrum were subjected to high-resolution tandem mass spectrometry (HRMS/MS) analysis. The *m*/*z* range for data-dependent acquisition was set between 150 and 2000 amu. HRMS/MS scans were obtained for selected ions with collision induced dissociation (CID) fragmentation, an isolation width of 2.0, a normalized collision energy of 35, an activation Q of 0.250, and an activation time of 30 ms. Data were analyzed using Thermo Xcalibur software (2.2 SP1 build 48).

### 2.4. LC-HRMS/MS Data Processing and Molecular Networking

Raw files were imported and pre-processed through MZmine 2.53 [12] software. First, a cropping of the raw data was performed narrowing the *m*/*z* range between 250 and 2000 amu. Mass detection involved processing cropped raw data and centroided masses at mass levels 1 and 2, maintaining the noise level at 10,000. Chromatograms were constructed using an ADAP module with a minimum height of 10,000 and *m*/*z* tolerance of 0.001 (or 5 ppm). Peak alignment employed the Join aligner algorithm with specified parameters: *m*/*z* tolerance at 0.005 (or 5 ppm) and absolute RT tolerance at 0.2 min. To enhance precision, [M+Na-H], [M+K-H], [M+Mg-2H], [M+NH_3_], [M+1, ^13^C], [M-^35^Cl+^37^Cl], [M+^56^Fe-3H] adducts were filtered out by setting the maximum relative height at 100%. Peaks lacking associated MS/MS spectra were subsequently excluded from the peak list. Feature-based data were exported to an .mgf file for GNPS, while chromatographic data, encompassing retention times, peak areas, and peak heights, were exported to a .csv file. A Feature-Based Molecular Network [13] was generated on GNPS’s online platform UCSD Computational Mass Spectrometry Website [14], with the following parameters: the precursor ion mass tolerance was established at 0.05 Da, while the MS/MS fragment ion tolerance was set to 0.05 Da. Criteria for acceptance included a cosine score surpassing 0.7 and a minimum of 6 matched peaks. Spectra were retained only if the nodes appeared in each other’s respective top 10 most similar nodes. In the GNPS library search, the criteria were defined as a cosine score of 0.7 and a minimum of 7 matched peaks. The visualization of the molecular network was accomplished using Cytoscape [15] software (3.10.1).

## 3. Results

### 3.1. Antioxidant Assay

The antioxidant activity of *Anabaena flos-aquae* was evaluated using four different assays, measuring total phenolic content (TPC), radical-scavenging activity (DPPH), reducing power (FRAP), and *β*-carotene bleaching assay (BCB) (Table 1).

Phenolic compounds, such as phenolic acids, tannins, and flavonoids are considered the greatest contributors to plants’ antioxidant activity. Thus, firstly, the total phenolic content and antioxidant activity of *Anabaena flos-aquae* fractions were evaluated using the Folin-Ciocalteau test (TPC). The TPC, in fact, is based on the electron transfer reaction, allowing the evaluation of the reductive antioxidant capacity of an extract. By using this test, it was demonstrated that the MeOH, CHCl_3_, and CHCl_3_/MeOH fractions showed a higher amount of phenolic compounds compared to the BuOH and water fractions (31.25 ± 2.67, 29.57 ± 1.11, 18.21 ± 0.31, 4.38 ± 0.28, 1.86 ± 0.28 mgGAE/g, respectively).

The antioxidant activity of *Anabaena flos-aquae* fractions was also evaluated using the FRAP assay since it was relevant to the TPC test. It is indeed known that phenols act as electron donors, thus reducing the ferric tripyridyltriazine complex to the ferrous complex [Fe(III)–TPTZ to Fe(II)–TPTZ]. Confirming the data from the TPC, the fraction with the higher FRAP value was the MeOH fraction (53.40 ± 1.72 mgGAE/g). However, in this case, CHCl_3_, CHCl_3_/MeOH, and BuOH fractions showed a non-significant difference in the obtained values (5.96 ± 0.29, 6.16 ± 0.53, 5.18 ± 0.33 mgGAE/g, respectively) while the aqueous ones demonstrated the lowest activity (0.73 ± 0.06 mgGAE/g).

The other assay used was the DPPH radical-scavenging assay based on the utilization of a free radical, the DPPH, which is able to accept an electron or hydrogen radical to transform itself into a stable diamagnetic molecule. In this study, the DPPH assay also corroborated the highest activity of the *Anabaena* MeOH fraction (17.89 ± 0.31 mgTE/g), which was followed by CHCl_3_, CHCl_3_/MeOH, and BuOH and water fractions (8.63 ± 0.71, 4.82 ± 0.34, 1.22 ± 0.06, 0.90 ± 0.06 mgTE/g, respectively).

Finally, to our knowledge, this is the first time the BCB test was used to evaluate *Anabaena flos-aquae* antioxidant activity. Specifically, the BCB evaluates the antioxidant’s ability to inhibit lipid peroxidation in either the initiation or propagation phase [16]. It was seen that only the MeOH and CHCl_3_/MeOH fractions were able to inhibit *β*-carotene bleaching (17.81 ± 0.97 and 41.73 ± 1.16 %AA), while no activity was seen for all other fractions.

**Figure 1:**
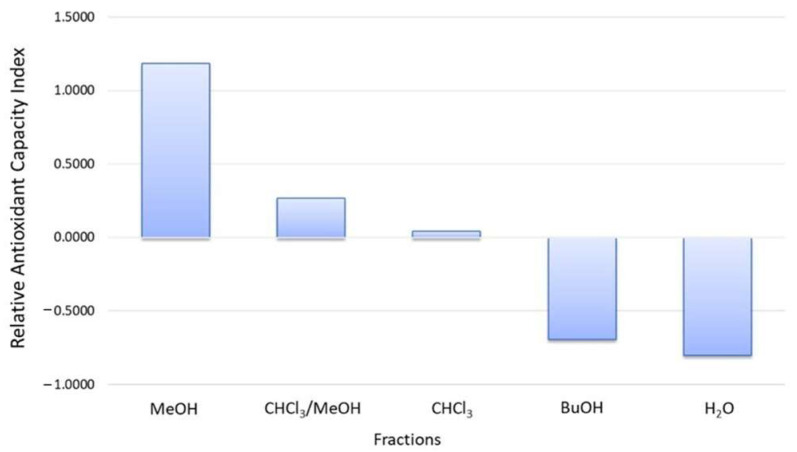
Relative Antioxidant Capacity Index (RACI) values were obtained comparing Total Phenolic Content (TPC), Ferric Reducing Antioxidant Power (FRAP), 2,2-diphenyl-1-picrylhydrazyl (DPPH), and *β*-carotene bleaching assay (BCB).

To compare the data obtained from all the antioxidant tests used and to validate the effective highest antioxidant activity of *Anabaena flos-aquae*’s MeOH extract, the Relative Antioxidant Capacity Index (RACI) was determined. The RACI is an adimensional and statistical index computed using Excel software (2010, Microsoft, Redmond, WA, USA) by comparing the value obtained from the evaluation of antioxidant activity with the different assays. This method validated that the MeOH fraction had the highest antioxidant activity with a value of 1.18, while those with the lowest activity were the BuOH and water fractions (Figure 1).

### 3.2. LC-HRMS Analyses and Molecular Networking

The *Anabaena flos-aquae* UTEX 1444 strain (sourced from Prof. A. Pollio’s collection) was cultivated in 0 µM Fe^3+^ BG11 [17] medium. Cultures—already known for their synechobactin production—were processed using our standard lab procedure [18]. In brief, all cultures were subjected to vortex, sonication, and subsequent centrifugation to separate solid pellets from the liquid supernatant. Then, the solid pellets underwent extraction using a combination of MeOH and CHCl_3_, whereas the supernatants were treated with BuOH. All extracted samples were then analyzed through liquid chromatography coupled with high-resolution mass spectrometry (LC-HRMS). The HRMS data underwent dereplication through molecular networking analysis, as detailed in prior reports [19].

The LC-HRMS analysis of all extracts from cultures with 0 µM Fe^3+^ was performed using an LTQ Orbitrap instrument. Data-dependent acquisition was employed to initiate MS2 scans for the five most intense ions detected in the full MS scan.

The antioxidant assays highlighted the MeOH fraction as the most active, with a RACI value of 1.18, while the BuOH and water fractions exhibited the lowest activity. Consequently, subsequent data processing and molecular networking focused on the methanol and butanol extracts comparing their metabolic profiles to target the compounds responsible for the activity. The raw LC-MS data underwent preprocessing using the MZmine program version 2.53 [12]. This step facilitated the elimination of isotopic peaks, the identification of adducts, and enabled quantitation. The comprehensive .mgf file, encompassing MS2 data from the methanol and butanol extracts along with the quantitation table obtained through mzMine, was submitted to the Global Natural Products Social Molecular Networking website. This platform generated a Feature-Based Molecular Network (FBMN) (UCSD Computational Mass Spectrometry Website), which was visualized using the Cytoscape program version 3.9.0 [15].

The resultant qualitative-quantitative network of methanol and butanol extracts comprises 433 features organized into 11 clusters. Within the network, each node is depicted as a pie chart, illustrating the compound’s abundance in the BuOH and MeOH extracts.

**Figure 2:**
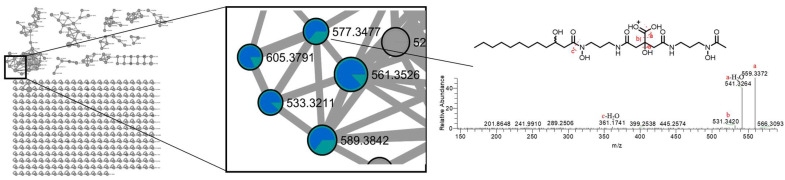
Molecular network and HRMS/MS analysis of MeOH and BuOH extracts of cultures of *Anabaena flos-aquae* UTEX 1444. Portion of cluster 1 belonging to synechobactins. Nodes are represented as a pie chart showing the source culture of the compound (BuOH extract (blue), MeOH extract (green)). Node size is proportional to metabolite amounts (precursor ion intensity). HRMS/MS analysis and fragmentation pattern of the new synechobactin OHC12. Synechobactins’variants are preferentially concentrated in BuOH extract.

Within the eleven clusters identified, one cluster stands out (Cluster 1), comprising 32 nodes, with 5 nodes associated with the synechobactins series, predominantly present in the BuOH extract. Notably, four of these nodes have been previously documented in our earlier publication [5]. The remaining node at *m*/*z* 577.3475 (**1**) ([M+H]^+^ C_26_H_49_O_10_N_4_^+^) has been identified as the novel synechobactin OHC_12_, characterized by an hydroxy acyl (dodecanoyl) chain. This new compound exhibits a distinctive fragment ion at *m*/*z* 361.1741 ([M+H-RCOH-H_2_O]^+^, C_14_H_25_O_7_N_4_^+^), stemming from the detachment of the fatty acid unit and a water molecule, likely originating from the citrate moiety. This observation suggests that the modification distinguishing this new variant from others lies in the acyl chain (Figure 2).

The presence of this diagnostic fragment, consistently found in all synechobactins [20], provides unequivocal information about the molecular nature of the compound.

Furthermore, three supplementary fragments were identified, resulting from the removal of one or two water molecules (18.0105 amu, *m*/*z* [M+H]^+^ 559.3372, C_26_H_47_O_9_N_4_^+^ and 36.0213 amu, *m*/*z* [M+H]^+^ 541.3264, C_26_H_45_O_8_N_4_^+^) along with the elimination of a carboxyl residue (46.0055 amu, *m*/*z* [M+H]^+^ 531.3420, C_25_H_47_O_8_N_4_^+^).

**Figure 3:**
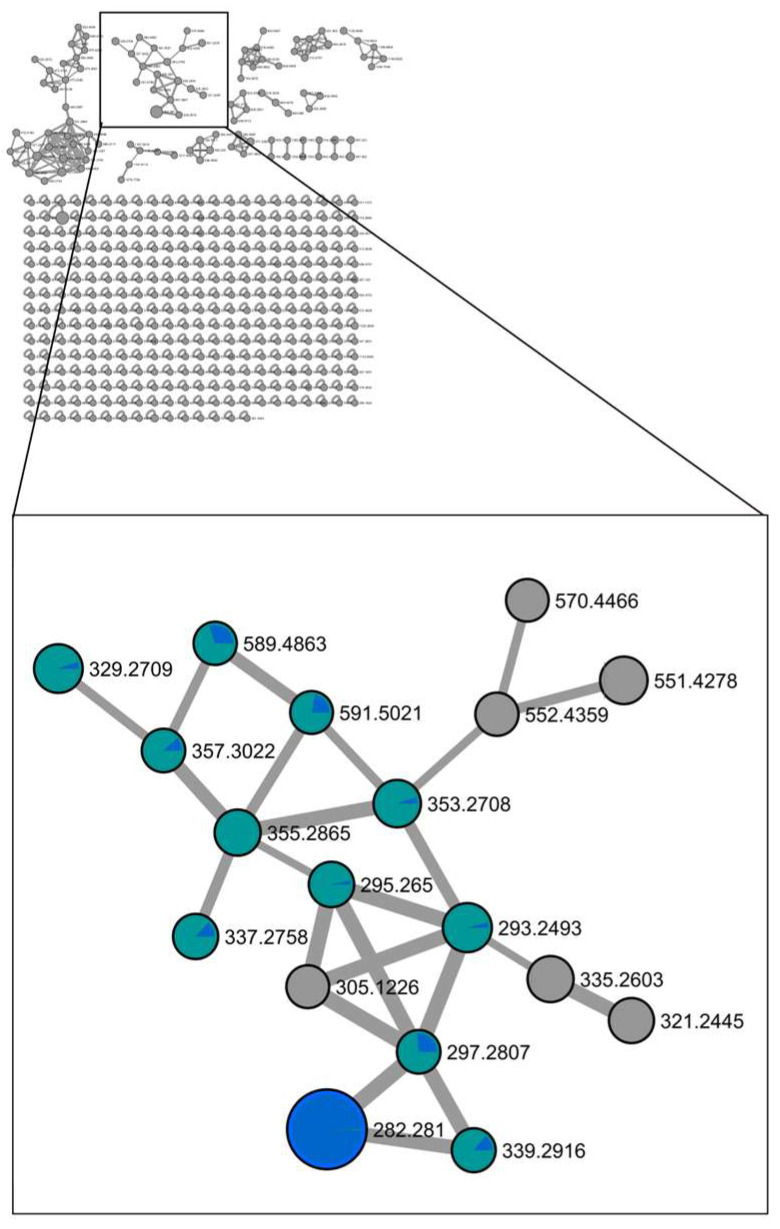
Cluster 2 of molecular network of MeOH and BuOH extracts of cultures of *Anabaena flos-aquae* UTEX 1444. Nodes are represented as a pie chart showing the source culture of the compound (BuOH extract (blue), MeOH extract (green). Node size is proportional to metabolite amounts (precursor ion intensity). Node in light grey, belonging to the MeOH extract, represent compounds that are not identified. Mono and diacylglycerols are preferentially concentrated in MeOH extract, the most active in antioxidant assays.

A second cluster (Cluster 2) encompasses 18 nodes, with 17 primarily associated with the highly active MeOH extract. Upon cluster analysis, the presence of monoacylglycerol (MG) and diacylglycerol (DG) compounds was revealed (Figure 3). Specifically, the compounds at *m*/*z* 357.3022 (**2**) ([M+H]^+^ C_21_H_41_O_4_^+^), *m*/*z* 355.2865 (**3**) ([M+H]^+^ C_21_H_39_O_4_^+^), and *m*/*z* 353.2708 (**4**) ([M+H]^+^ C_21_H_37_O_4_^+^) were confirmed as monoacylglycerols with octadecyl chains that were mono, di, and tri-unsaturated, respectively. Their structural confirmation was achieved through the analysis of the HRMS/MS spectra, revealing diagnostic fragment ions at *m*/*z* 265.2543 ([RCO]^+^ C_18_H_33_O^+^), *m*/*z* 263.2388 ([RCO]^+^ C_18_H_31_O^+^), and *m*/*z* 261.2231 ([RCO]^+^ C_18_H_29_O^+^), corresponding to the acylium ion generated from the neutral loss of the glycerol moiety (C_3_H_8_O_3_, 92.0473 Da). Moreover, the carbonyl group likely removes a proton from the terminal primary OH, thereby facilitating the elimination of a lactone ring and formation of another diagnostic fragment, i.e., [M+H-C_3_H_6_O_2_]^+^.

Similarly, the compound at *m*/*z* 329.2709 (**5**) ([M+H]^+^ C_19_H_37_O_4_^+^) was identified as a monacylglycerol with a monounsaturated hexadecyl chain using the same approach.

Analyzing the fragmentation patterns of the compounds present in the cluster, the nodes at *m*/*z* 339.2916 (**6**) ([M+H]^+^ C_21_H_39_O_3_^+^) and at *m*/*z* 337.2758 (**7**) ([M+H]^+^ C_21_H_37_O_3_^+^) were identified as monoacylglycerols. Specifically, compound **6** had an octadecyl chain that was monounsaturated, while compound **7** had an octadecyl chain that was di-unsaturated, both lacking an oxygen on the glycerol moiety (Table 2).

**Figure 4:**
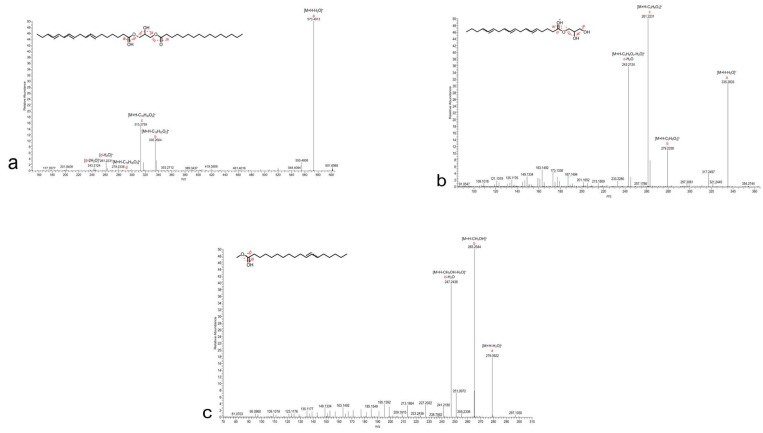
HRMS/MS analysis and fragmentation pattern of (**a**) compound **8** DG (18:3/16:0), (**b**) compound **4** MG18:3, and (**c**) compound **10** FAME 18:1.

Based on the fragmentation patterns, the node labeled with *m*/*z* 591.5021 (**8**) ([M+H]^+^ C_37_H_67_O_5_^+^) was identified as a diacylglycerol with a tri-unsaturated octadecyl chain and a fully saturated hexadecyl chain, while the node at *m*/*z* 589.4863 (**9**) ([M+H]^+^ C_37_H_65_O_5_^+^) was identified as a diacylglycerol with a tri-unsaturated octadecyl chain and a monounsaturated hexadecyl chain (Figure 4).

The compounds at *m*/*z* 297.2807 (**10**) ([M+H]^+^ C_19_H_37_O_2_^+^), *m*/*z* 295.2650 (**11**) ([M+H]^+^ C_19_H_35_O_2_^+^) and *m*/*z* 293.2495 (**12**) ([M+H]^+^ C_19_H_33_O_2_^+^), were determined to be the methyl esters of fatty acids with octadecyl chains and one, two and three unsaturations, respectively. Their structures were confirmed through the analysis of the HRESIMS/MS spectra, revealing the subsequent loss of MeOH and a water residue for each. HRESIMS/MS spectra of all compounds are reported in Supplementary Materials.

The presence of these molecules, unsaturated mono and diacylglycerols particularly in the highly active MeOH extract, suggests a potential correlation between their presence and the observed activity [21,22,23].

In addition, the presence in this cluster of a compound at *m*/*z* 282.2810 (**13**) ([M+H]^+^ C_18_H_36_ON^+^) corresponding to an octadecyl amide with only one unsaturation mainly found in the not active BuOH extract is in line with the general understanding that compounds with unsaturated structures, such as those containing double bonds, are often associated with antioxidant properties. In the context of antioxidant nutraceutical compounds, unsaturated compounds can be effective because they are capable of donating electrons to neutralize free radicals. Free radicals, which are highly reactive molecules with unpaired electrons, can cause oxidative stress and damage to cells. Antioxidants help counteract this damage by donating electrons to stabilize free radicals, then considered very effective as antiaging nutraceuticals, as dietary supplements, or functional foods.

## 4. Discussion

Antioxidant nutraceutical compounds are of pivotal importance in the realm of health and wellness. Antioxidants play a crucial role in neutralizing harmful free radicals in the body, which are reactive molecules that can cause oxidative stress. Oxidative damage triggered by reactive oxygen species (ROS) on nucleic acids, lipids, and proteins can be the cause of various chronic disease outbreaks, such as cancer, atherosclerosis, neurological diseases, coronary heart diseases, and ageing. ROS are also the major responsible for lipid-containing food degradation, which makes it necessary to add synthetic antioxidants to food. However, in the last years, several investigations demonstrated that synthetic antioxidants generally used by food industries, such as butylated hydroxytoluene (BHT), tert-butylhydeoquinone (TBHQ), and butylated hydroxyanisole (BHA), are not without side effects for human health [24]. For this reason, the research of a new safe antioxidant from natural origin is demanded. Natural sources of antioxidants, such as those found in fruits, vegetables, and certain microorganisms, such as cyanobacteria, are particularly valuable. Incorporating antioxidant-rich substances into one’s diet or utilizing them in various applications, such as in the development of nutraceuticals, represents a proactive approach to supporting health and preventing the adverse effects associated with oxidative stress. Nitrogen-fixing (diazotrophic) cyanobacteria can represent an interesting source of antioxidant compounds since, in these microalgae, the defense against ROS is particularly important for the occurrence of N-fixation [25]. In fact, the intrinsic conflict between the fixation of nitrogen and the evolution of photosynthetic oxygen in diazotrophic cyanobacteria has prompted the development of several protective mechanisms against ROS and oxygen. Among these mechanisms, cyanobacteria have developed several biosynthetic pathways, such as the shikimate pathway, to produce specialized metabolites involved in reducing free radicals or decreasing their production rate [26]. Based on this background, it was decided to investigate the *Anabaena flos-acquae*’s ability to counteract oxidative stress.

It is widely understood that the antioxidant activity of an extract cannot be fully assessed by a single test because antioxidants are a complex class of molecules, each of which can counteract specific radicals or oxidizers based on their chemical structures. Consequently, employing multiple techniques becomes imperative to grasp the full spectrum of an extract’s antioxidant traits [7,27]. In the present investigation, four different assays, TPC, DPPH, FRAP, and BCP, were used to evaluate the antioxidant activity of each *Anabaena flos-acquae*’s fraction. Using the TPC assay, it was seen that the amount of phenolic compounds determined in MeOH, CHCl_3_, and CHCl_3_/MeOH fractions was higher than that described in another study where the partition was achieved with another polar solvent, ethyl acetate [28]. In this case, in fact, the *Anabaena flos-aquae* showed a TPC value of 2.99 ± 0.31 mgGAE/g, indicating that MeOH and CHCl_3_ should be more effective in extracting phenolic compounds than ethyl acetate [29]. Confirming data from TPC, the fraction with the higher FRAP and DPPH value was the MeOH fraction. *Anabaena flos-aquae* antioxidant activity was also evaluated with FRAP assay in other investigations. Nonetheless, the data are not comparable due to the use of standards other than gallic acid, such as ascorbic acid [30,31]. A validation of the highest antioxidant activity of the MeOH fraction comes from the evaluation of the RACI; hence, this fraction was further investigated for its metabolomic content.

In the present work, a sustainable strategy to study the metabolomic contents of biomass extracts revealed the presence of unsaturated mono and diacylglycerols as the main components of the most active MeOH extract of *Anabaena flos-aquae*, which suggests them as responsible for the antioxidant activity observed.

Indeed, unsaturated mono and diacylglycerols, characterized by the presence of at least a double bond in their acyl chains, have emerged as noteworthy antioxidant compounds with potential health benefits [21,22,23]. These molecules, found in various natural sources, such as certain oils and plant extracts, exhibit distinctive antioxidant properties attributable to their specific chemical structure. The double bonds are a key feature that contributes to their antioxidant activity. Unsaturated fatty acids, forming the acyl groups, possess the ability to donate electrons, making them effective scavengers of free radicals and reactive oxygen species (ROS). By donating electrons to stabilize radicals, these compounds help prevent cellular damage and mitigate the risk of oxidative stress-related diseases. The specific arrangement of carbon atoms and the degree of unsaturation in the acyl chains influence the antioxidant efficacy of unsaturated diacylglycerols. Polyunsaturated fatty acids (PUFAs), characterized by multiple double bonds, are particularly recognized for their strong antioxidant effects. Like unsaturated triglycerides, unsaturated diacylglycerols contribute to the protection of cell membranes against lipid peroxidation. This is particularly important for maintaining the integrity and functionality of cell membranes, as lipid peroxidation can compromise these vital cellular structures. Beyond their direct antioxidant effects, unsaturated diacylglycerols exhibit anti-inflammatory properties. Inflammation is closely linked to oxidative stress and various chronic diseases. By modulating inflammatory responses, these compounds contribute to an overall reduction in oxidative burden. Research suggests that diets rich in unsaturated diacylglycerols, often derived from sources such as olive oil, certain nuts, and seeds, may confer health benefits. These include cardiovascular protection, support for cognitive health, and a reduced risk of chronic diseases associated with oxidative stress.

In summary, unsaturated diacylglycerols represent a valuable class of antioxidant compounds with the potential to positively impact human health. Their ability to combat oxidative stress through radical scavenging, membrane protection, and anti-inflammatory mechanisms underscores their significance in the context of preventive and therapeutic approaches. Ongoing research continues to explore and unveil the diverse health-promoting properties of these compounds.

## Figures and Tables

**Table 1:** Antioxidant activity of *Anabaena flos-aquae* fractions.

	TPC ^1^	DPPH ^2^	FRAP ^3^	BCB ^4^
	mgGAE/g	mgTE/g	mgGAE/g	%AA
**MeOH**	31.25 ± 2.67 ^a^	17.89 ± 0.31 ^a^	53.40 ± 1.72 ^a^	17.81 ± 0.97 ^b^
**CHCl_3_**	29.57 ± 1.11 ^a^	8.63 ± 0.71 ^b^	5.96 ± 0.29 ^b^	0
**CHCl_3_/MeOH**	18.21 ± 0.31 ^b^	4.82 ± 0.34 ^c^	6.16 ± 0.53 ^b^	41.73 ± 1.16 ^a^
**BuOH**	4.38 ± 0.28 ^c^	1.22 ± 0.06 ^d^	5.18 ± 0.33 ^b^	0
**H_2_O**	1.86 ± 0.10 ^d^	0.90 ± 0.06 ^d^	0.73 ± 0.06 ^c^	0

^1^ TPC: total phenolic content in milligrams of gallic acid per gram of extract; ^2^ DPPH: 2,2-diphenyl-1-picrylhydrazyl in milligrams of Trolox equivalents per gram; ^3^ FRAP: ferric reducing antioxidant power in milligrams of Trolox equivalents per grams of extract; ^4^ BCB: *β*-carotene bleaching assay calculated as follows: (A*β*-carotene after 180 min/A initial *β*-carotene) × 100 (AA%). The experiments were conducted three times, and the results are presented as mean ± standard deviation (SD). Significant differences (*p* < 0.05) are denoted by distinct letters (a–d).

**Table 2:** Acylglycerol analogues in cluster 2.

n.	RT	Annotation	*m*/*z* [M+H]^+^	Formula
**2**	33.5	MG 18:1	357.3022	C_21_H_41_O_4_^+^
**3**	32.5	MG 18:2	355.2865	C_21_H_39_O_4_^+^
**4**	31.5	MG 18:3	353.2708	C_21_H_37_O_4_^+^
**5**	31.8	MG 16:1	329.2709	C_19_H_37_O_4_^+^
**6**	33.5	MGlycidol 18:1	339.2916	C_21_H_39_O_3_^+^
**7**	32.5	MGlycidol 18:2	337.2758	C_21_H_37_O_3_^+^
**8**	38.2	DG (18:3/16:0)	591.5021	C_37_H_67_O_5_^+^
**9**	37.8	DG (18:3/16:1)	589.4863	C_37_H_65_O_5_^+^
**10**	36.3	FAME 18:1	297.2807	C_19_H_37_O_2_^+^
**11**	35.5	FAME 18:2	295.2650	C_19_H_35_O_2_^+^
**12**	34.8	FAME 18:3	293.2495	C_19_H_33_O_2_^+^

## Data Availability

Data are contained within the article and Supplementary Materials.

## Supplementary Materials

The following supporting information can be downloaded at: https://www.mdpi.com/article/10.3390/microorganisms12010175/s1. Figure S1. MS/MS spectrum of compound **1** *m*/*z* 577.3475 synechobactin OHC12; Figure S2. MS/MS spectrum of compound **2** *m*/*z* 357.3022 MG 18:1; Figure S3. MS/MS spectrum of compound **3** *m*/*z* 355.2865 MG 18:2; Figure S4. MS/MS spectrum of compound **4** *m*/*z* 353.2708 MG 18:3; Figure S5. MS/MS spectrum of compound **5** *m*/*z* 329.2709 MG 16:1; Figure S6. MS/MS spectrum of compound **6** *m*/*z* 339.2916 MGlycidol 18:1; Figure S7. MS/MS spectrum of compound **7** *m*/*z* 337.2758 MGlycidol 18:2; Figure S8. MS/MS spectrum of compound **8** *m*/*z* 591.5021 DG (18:3/16:0); Figure S9. MS/MS spectrum of compound **9** *m*/*z* 589.4863 DG (18:3/16:1); Figure S10. MS/MS spectrum of compound **10** *m*/*z* 297.2807 FAME 18:1; Figure S11. MS/MS spectrum of compound **11** *m*/*z* 295.2650 FAME 18:2; Figure S12. MS/MS spectrum of compound **12** *m*/*z* 293.2495 FAME 18:3; Figure S13. Molecular network of MeOH and BuOH extracts of *Anabaena flos-aquae* UTEX 1444.
