# Supplementary material for: Harnessing Cyanobacteria’s Bioactive Potential: A Sustainable Strategy for Antioxidant Production
Source: Microorganisms. 2024 Jan 16;12(1):175. doi: 10.3390/microorganisms12010175 (PMC10820942; doi:10.3390/microorganisms12010175)
Supplement: Supplementary file 1 [file microorganisms-12-00175-s001.zip › microorganisms-2811957-supplementary.pdf]

Supplementary information

Harnessing Cyanobacteria's Bioactive Potential: A Sustainable Strategy for Antioxidants' Production

Teresa De Rosa [1,†], Maria Ponticelli [2,†], Roberta Teta [1], Vittorio Carlucci [2], Luigi Milella [2], Germana Esposito [1,*] and Valeria Costantino [1,*]

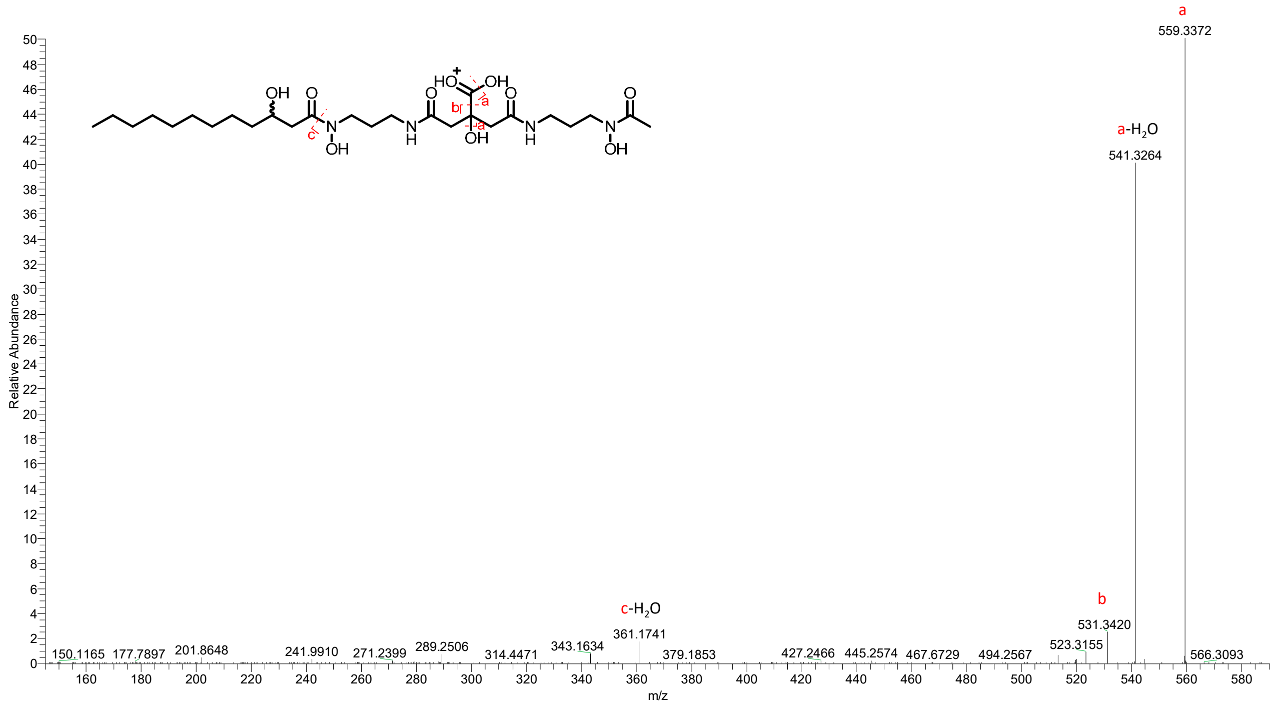

Figure S1. MS/MS spectrum of compound **1** m/z 577.3475 synechobactin OHC12

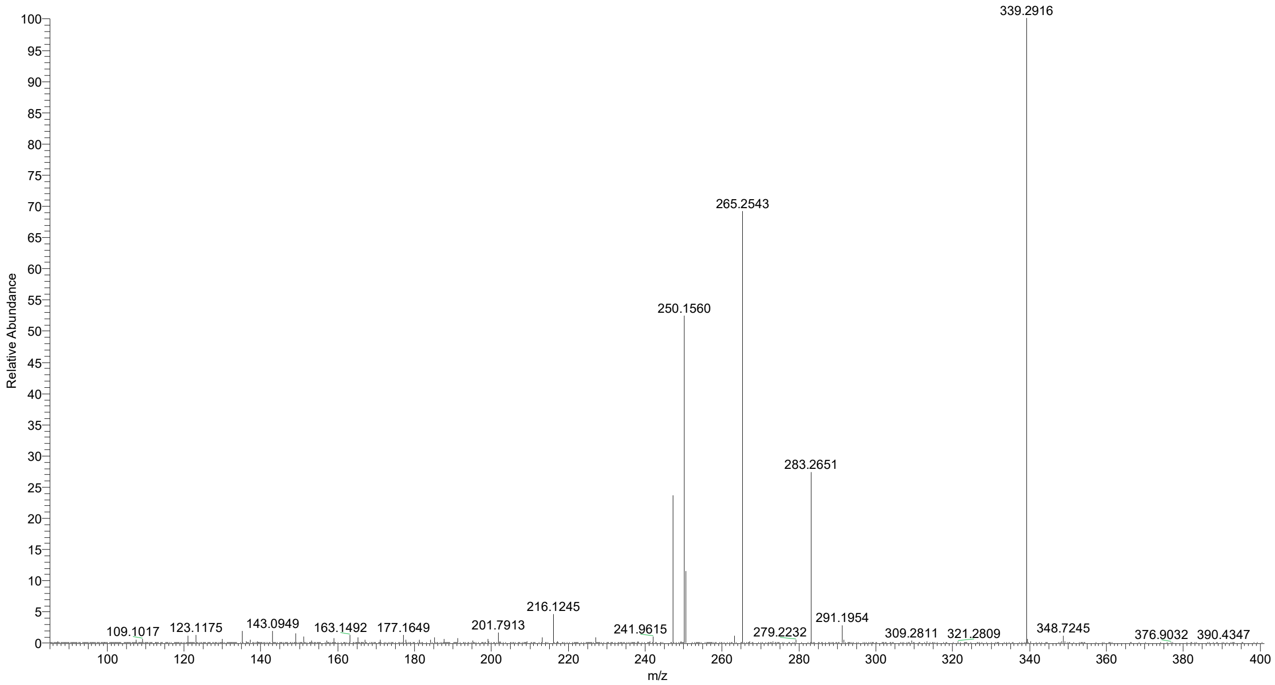

Figure S2. MS/MS spectrum of compound **2** m/z 357.3022 MG 18:1

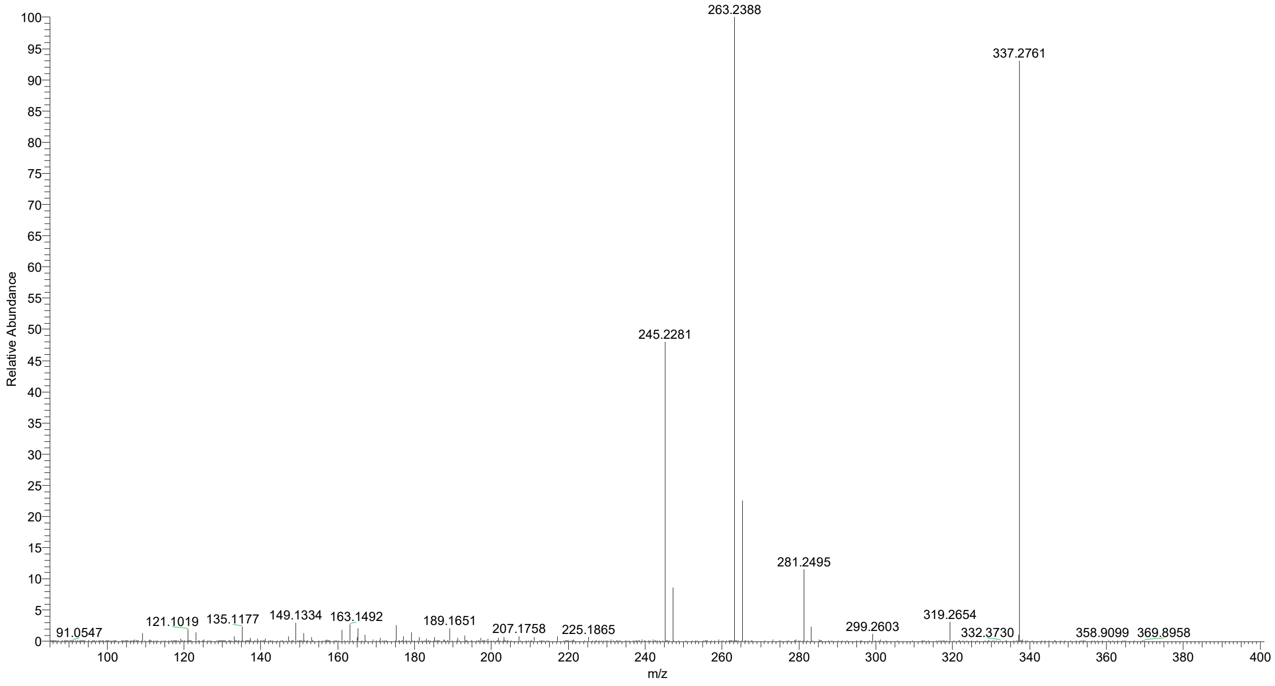

Figure S3. MS/MS spectrum of compound **3** m/z 355.2865 MG 18:2

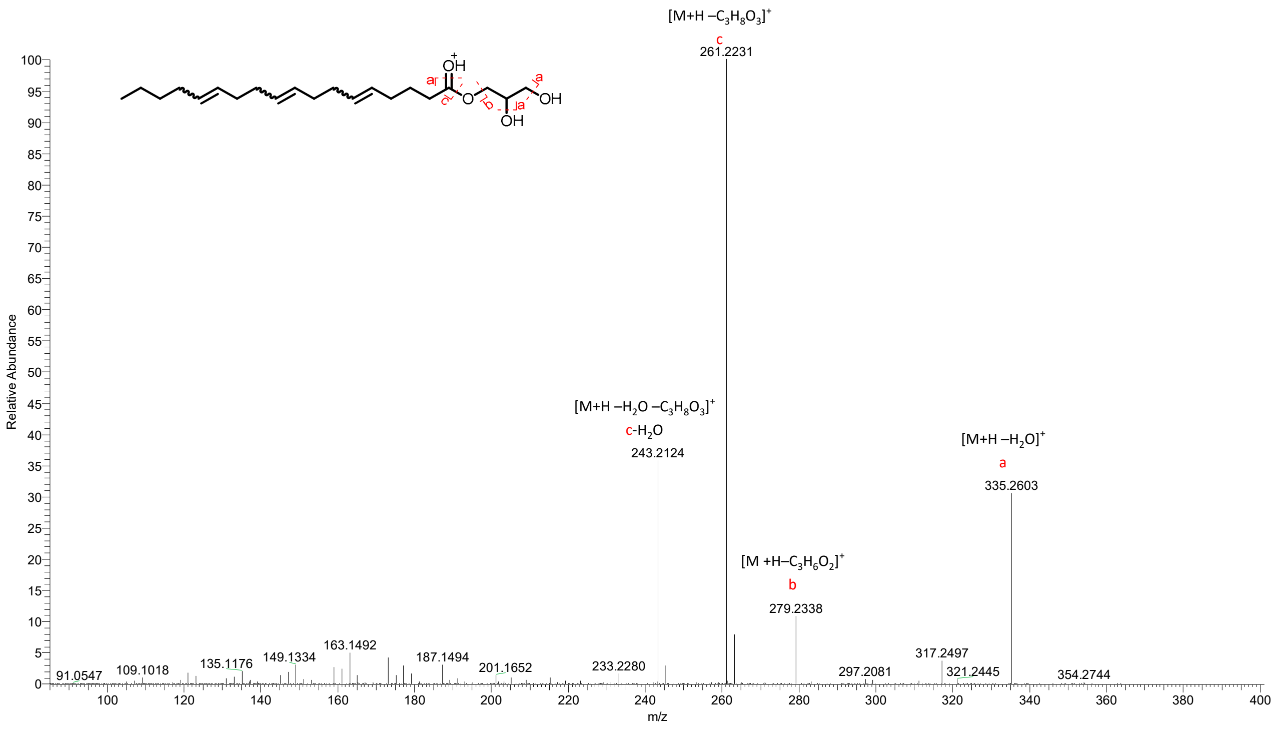

Figure S4. MS/MS spectrum of compound **4** m/z 353.2708 MG 18:3

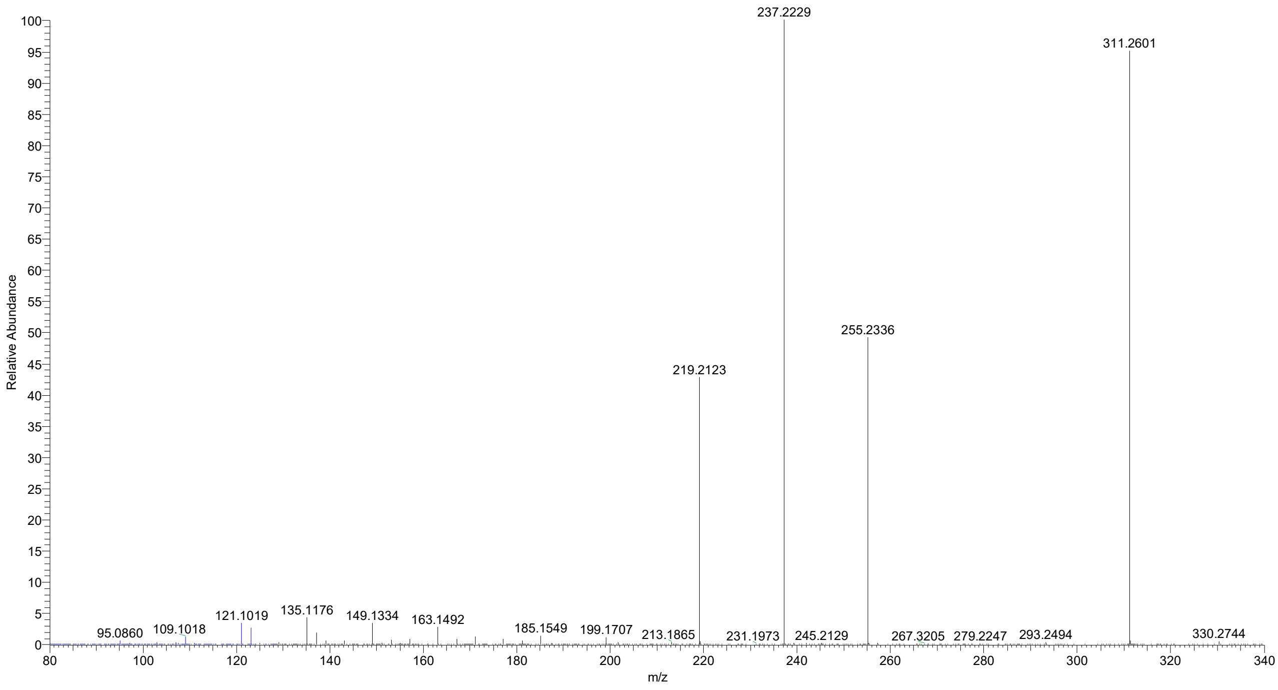

Figure S5. MS/MS spectrum of compound **5** m/z 329.2709 MG 16:1

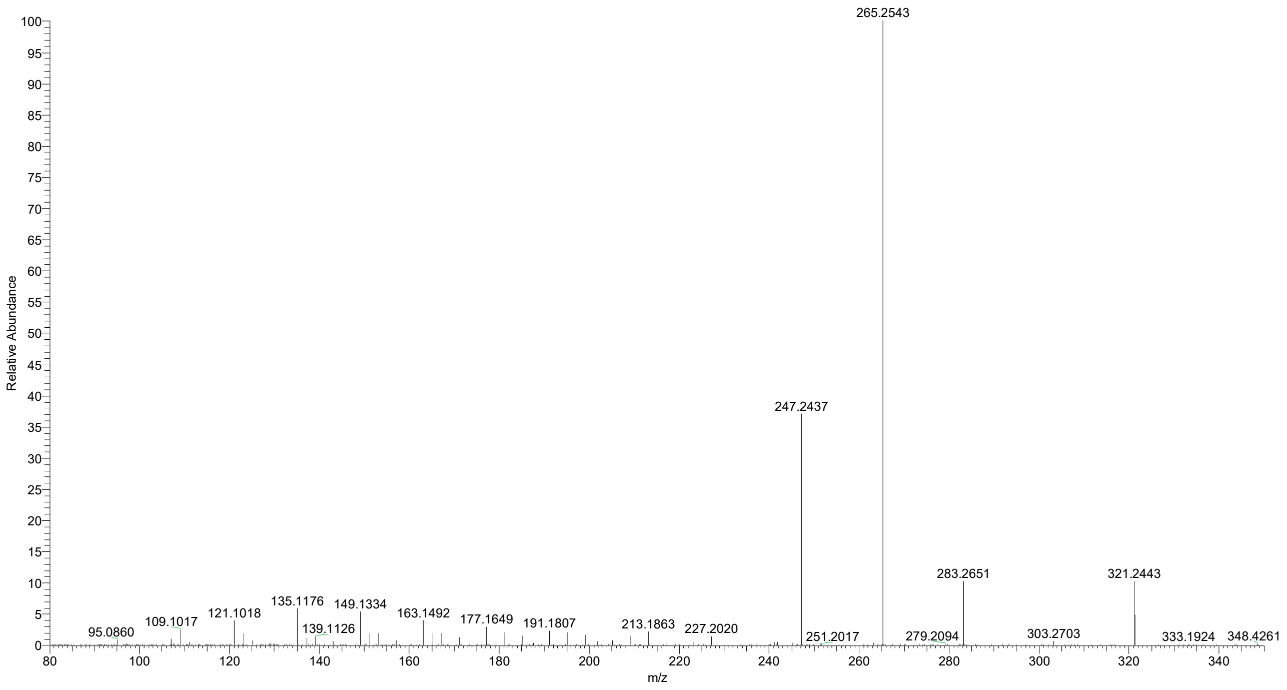

Figure S6. MS/MS spectrum of compound **6** m/z 339.2916 MGlycidol 18:1

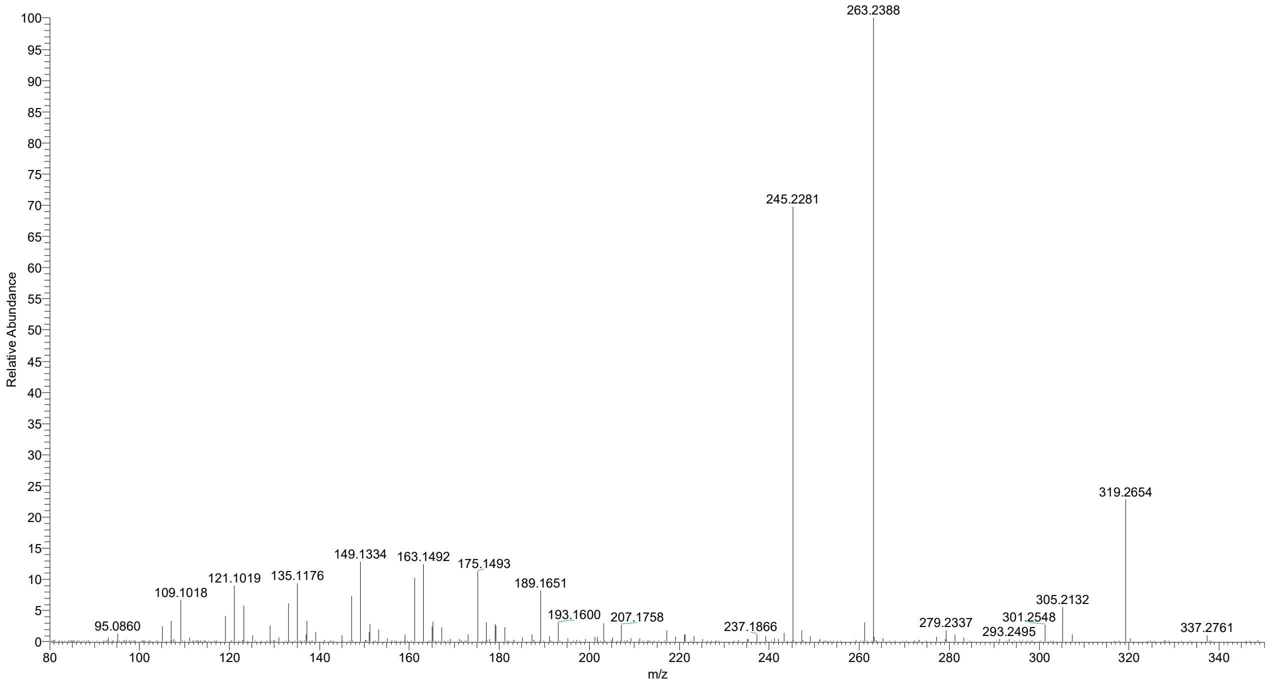

Figure S7. MS/MS spectrum of compound **7** m/z 337.2758 MGlycidol 18:2

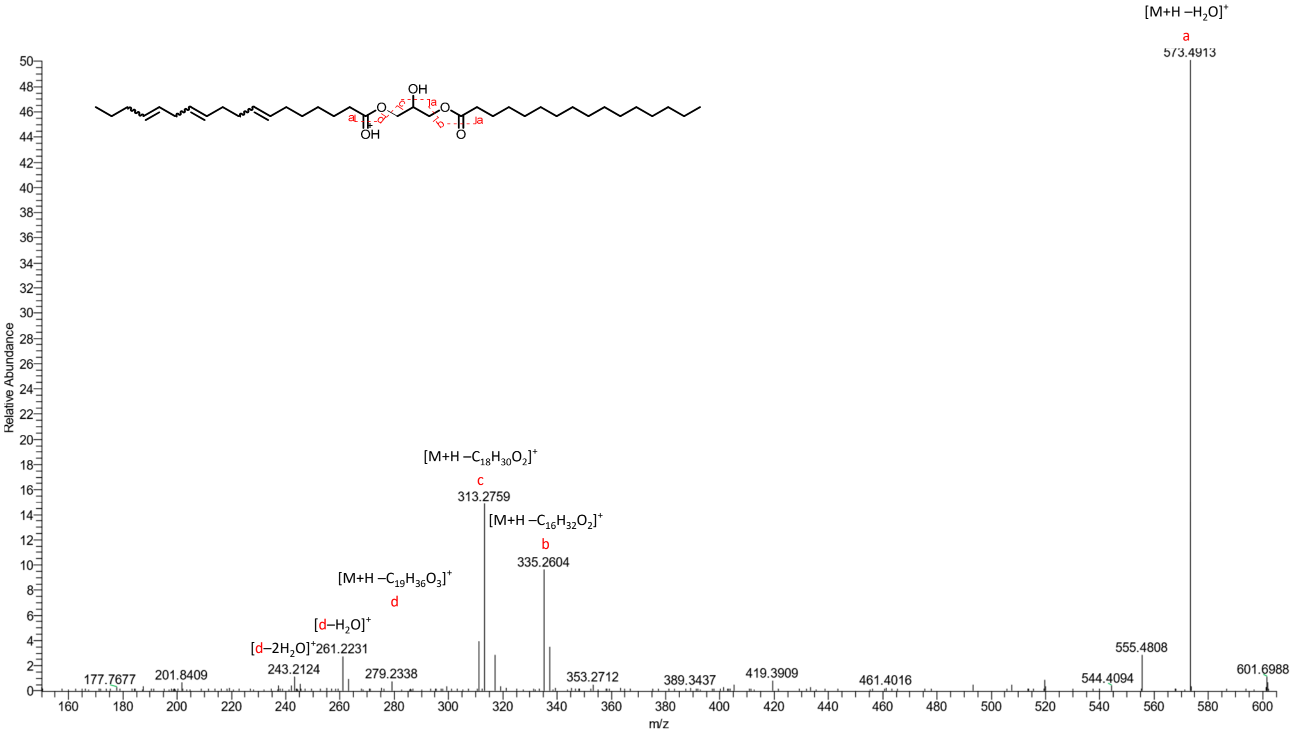

Figure S8. MS/MS spectrum of compound **8** m/z 591.5021 DG (18:3/16:0)

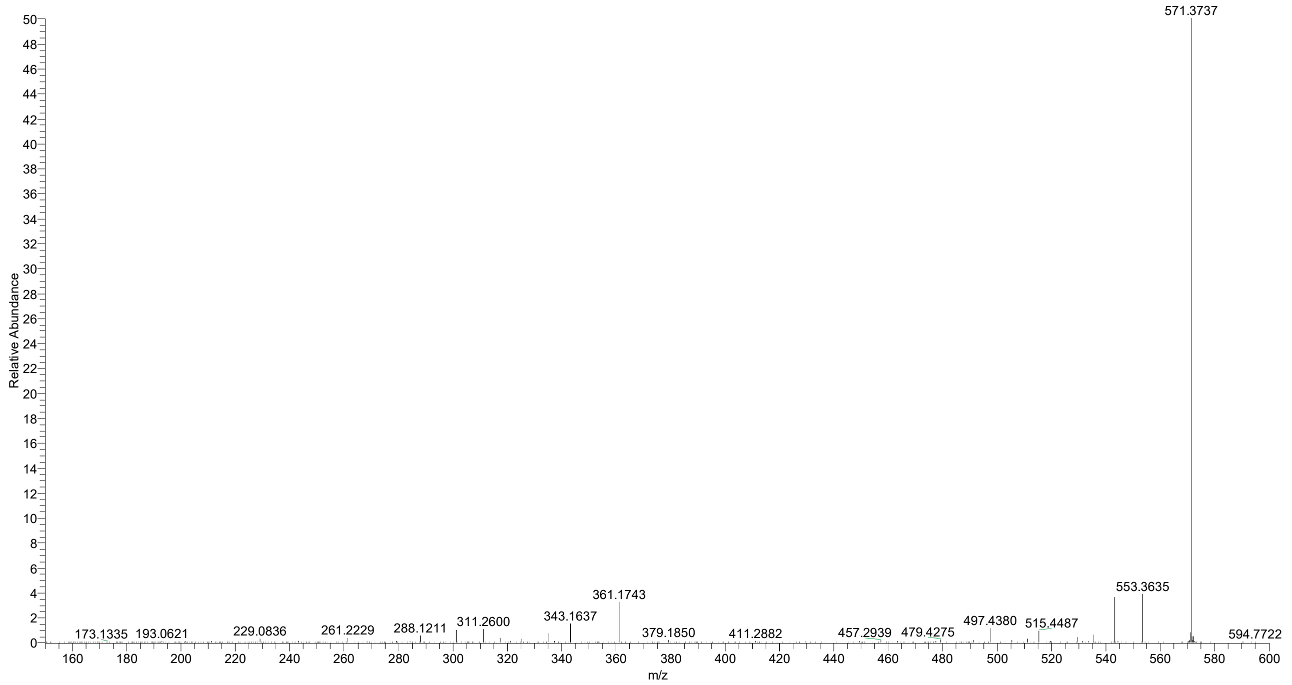

Figure S9. MS/MS spectrum of compound **9** m/z 589.4863 DG (18:3/16:1)

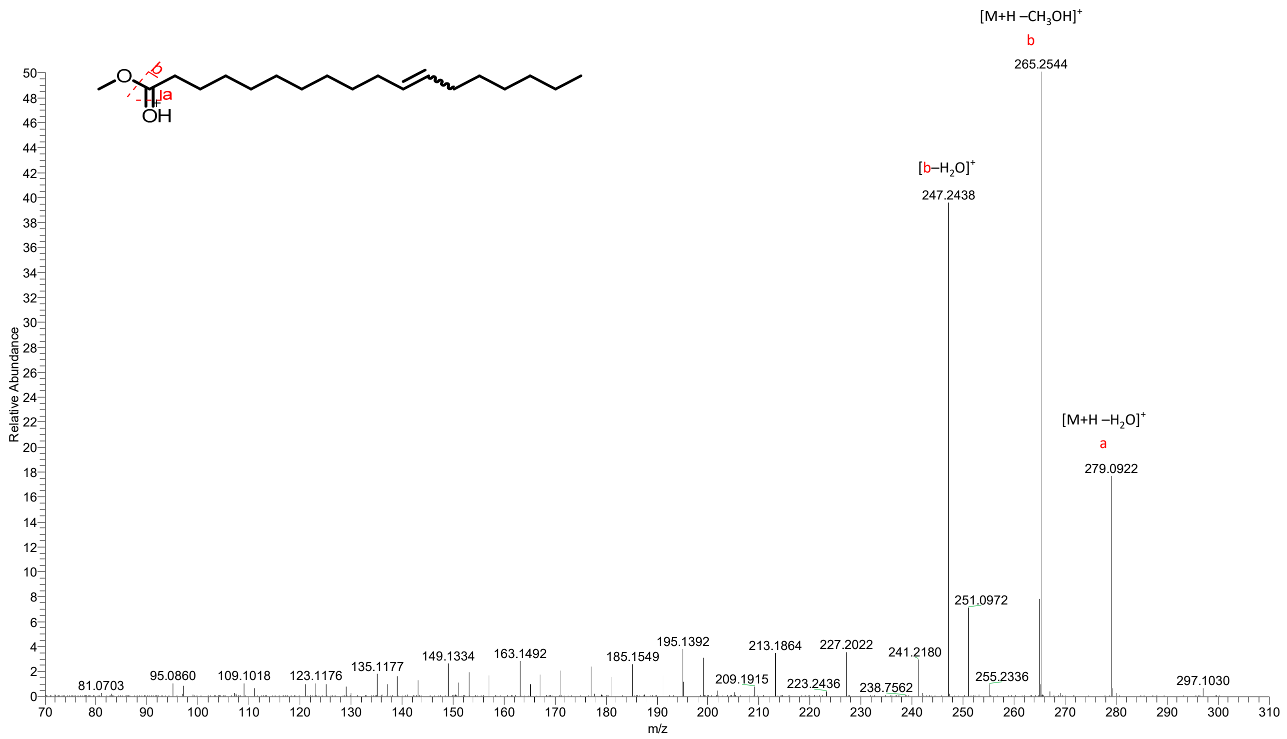

Figure S10. MS/MS spectrum of compound **10** m/z 297.2807 FAME 18:1

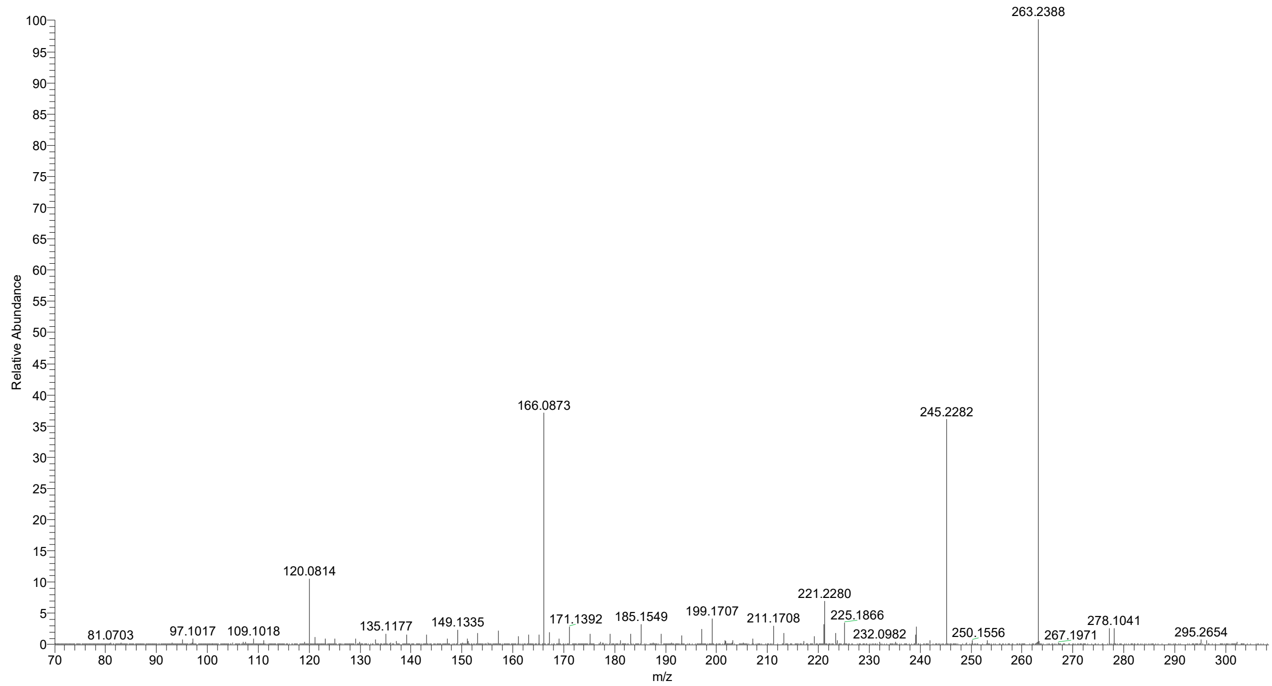

Figure S11. MS/MS spectrum of compound **11** m/z 295.2650 FAME 18:2

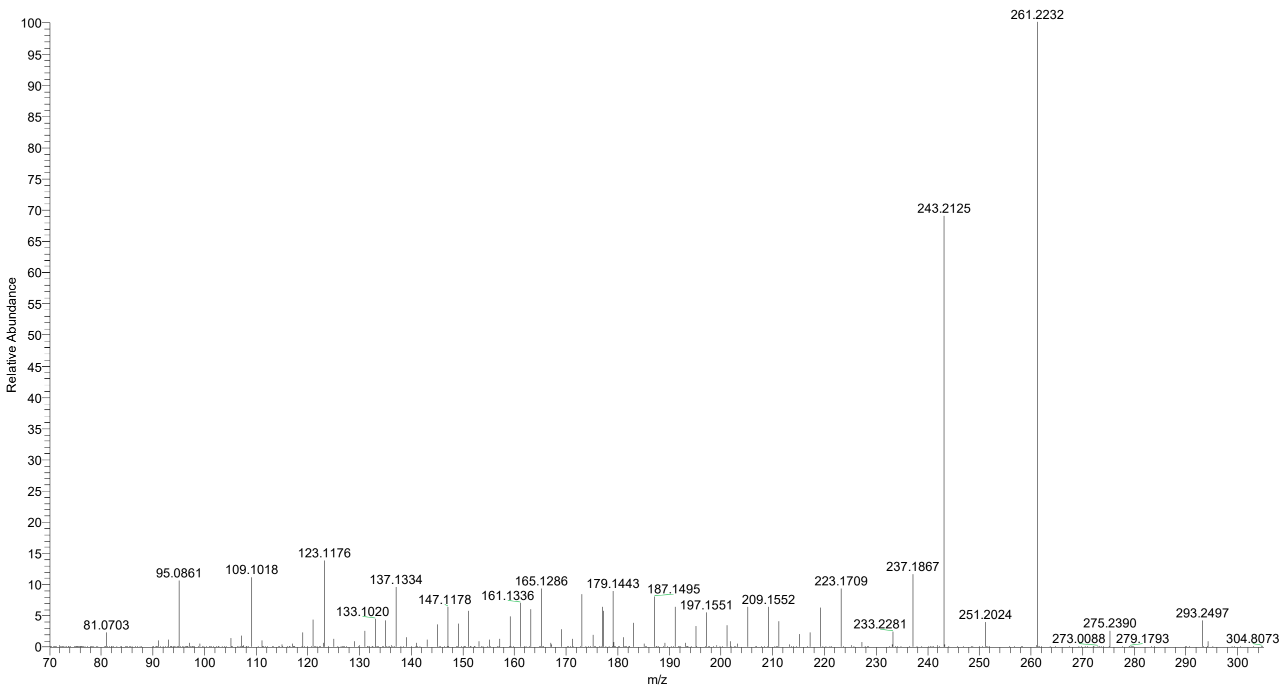

Figure S12. MS/MS spectrum of compound **12** m/z 293.2495 FAME 18:3

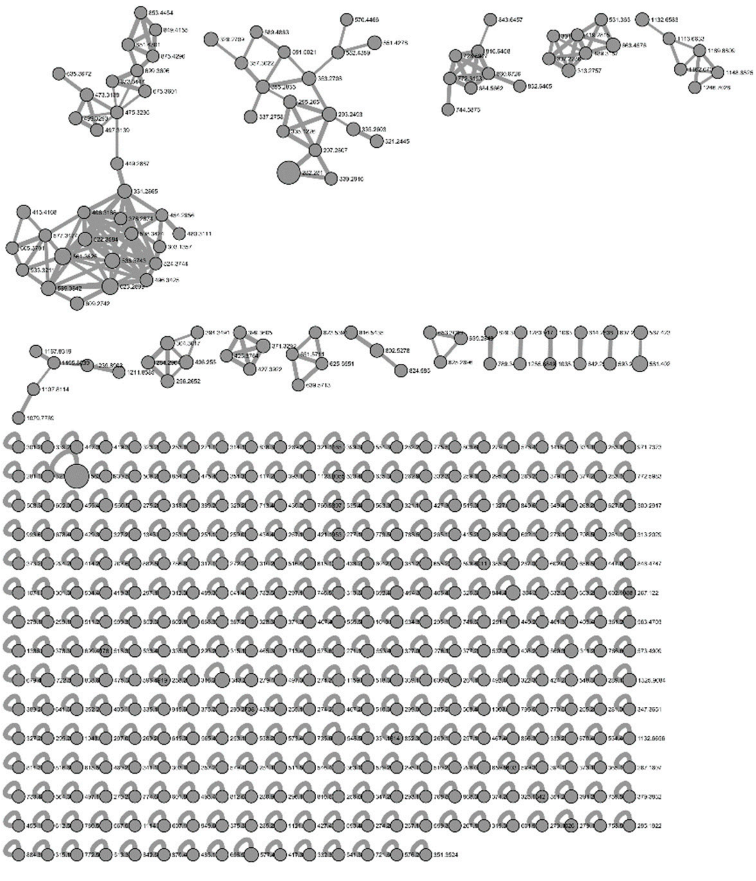

Figure S13. Molecular network of MeOH and BuOH extracts of *Anabaena flos-aquae* UTEX 1444
